# Physical Aspects of Viral Membrane Fusion

**DOI:** 10.1100/tsw.2009.76

**Published:** 2009-08-11

**Authors:** Laura Wessels, Keith Weninger

**Affiliations:** Department of Physics, North Carolina State University, Raleigh, USA

**Keywords:** viral membrane fusion; class I, II, III fusion proteins; hemagluttin (HA); E1; gp64

## Abstract

Enveloped viruses commonly employ membrane fusion during cell penetration in order to deliver their genetic material across the cell boundary. Large conformational changes in the proteins embedded in the viral membrane play a fundamental role in the membrane fusion process. Despite the tremendously wide variety of viruses that contain membranes, it appears that they all contain membrane fusion protein machinery with a remarkably conserved mechanism of action. Much of our current biochemical understanding of viral membrane fusion has been derived from high-resolution structural studies and solution-based *in vitro* assays in which viruses fuse with liposomes or cells. Recently, single-particle experiments have been used to provide measurements of details not available in the bulk assays. Here we focus our discussion on the key dynamical aspects of fusion protein structure, along with some of the experimental and computational techniques presently being used to investigate viral-mediated membrane fusion.

## INTRODUCTION

Membrane fusion is a vital phenomenon that allows eukaryotic cells to transfer materials among their membrane-encased compartments. Biological membranes do not spontaneously fuse under normal physiological conditions, so proteins are critical to mediate biological instances of membrane fusion. Eukaryotic cells have a highly conserved system of proteins known as SNAREs (soluble N-ethylmaleimide-sensitive factor [NSF] attachment protein [SNAP] receptors) that control intracellular membrane fusion[1]. Fertilization, fusion of mitochondria, and cell-cell fusion processes during development each use distinct proteins for membrane fusion[2,3,4]. One of the most easily studied biological systems involving membrane fusion is infection of a cell by an enveloped virus[5,6,7]. Enveloped viruses commonly release their genomes into a cell by fusing with the plasma membrane or by fusing from within an endosome following endocytosis.

All instances of protein-mediated membrane fusion require surmounting significant kinetic and energetic barriers, and membrane fusion proteins allow these barriers to be overcome. The prefusion states of membrane fusion proteins are metastably positioned away from the global free-energy minimum. These proteins mediate fusion by a process involving large conformational changes to lower energy states[7,8,9,10].

The transition from the metastable state to the lower energy state for viral fusion proteins is triggered when a virus encounters a specific cue from a targeted cell. Enveloped viruses that are endocytosed by cells use the endosomal acidification to trigger the conformational changes that lead to membrane fusion. Other viruses are triggered to fuse with the plasma membrane of cells by other signals, e.g., coreceptor binding. In either case, the membrane remodeling mechanisms used by all viral fusion proteins to mediate fusion appear universal. First, the proteins change into a more extended conformation that allows the exposure of a hydrophobic fusion peptide that is initially buried within the untriggered protein. This extended conformation allows the fusion peptide to insert and bury itself into an opposing membrane. Viral membrane fusion proteins are anchored in the virus membrane by transmembrane domains. Thus, this extended, fusion-activated state, with the fusion peptide in the target membrane, cross-links the two membranes that are destined to fuse. The fusion protein then folds back on itself into a hairpin configuration, bringing the two apposing membranes into near contact. A series of intermediate states is thought to form at this point, involving various lipidic structures, including a hemifusion stalk, a single bilayer diaphragm, and a fusion pore. The free energy of these intermediate structures depends on their geometry, the properties of the lipids in the membranes, the interactions of the lipids with the transmembrane domain, and the fusion peptide of the proteins, and may be affected by cooperativity between multiple fusion proteins[11,12,13]. The opening of a fusion pore allows the viral capsid access to the interior of the cell where it can potentially initiate infection. X-ray crystallography, electron microscopy, biochemical assays, genetic studies, computational/theoretical modeling, and fluorescence microscopy have all contributed to this general picture of the molecular events during viral membrane fusion.

## FUSION PROTEIN STRUCTURE

Viral membrane fusion proteins share some common features. All are integral membrane proteins with transmembrane domains that anchor them in the viral envelope. Viral fusion proteins often include domains inside the virus particle that are adjacent to the transmembrane domain. These internal domains are typically important for virus particle assembly, but appear to play no significant role in membrane fusion. The larger, external domains of the viral fusion proteins naturally separate into three classes based on common structural paradigms, leading to their classifications as class I, class II, or class III (described below and see Table 1). Regardless of the class, a common feature of the external domains is the presence of a short sequence of hydrophobic amino acids. This region is critical for membrane fusion activity and is called the fusion peptide.

**TABLE 1**
**Properties of Class I, II, and III Fusion Proteins**

| | Class I | Class II | Class III |
|---|---|---|---|
| Secondary structure of fusion proteins[12] | Mainly α-helical | Mainly composed of β-sheets | Mixed secondary structure |
| Location of fusion peptide[13] | Usually located near N-terminus | Located internally | Located internally |
| Postfusion structure[13] | Trimer of hairpins | Trimer of hairpins | Trimer of hairpins |

## Class I Fusion Proteins

The class I proteins are characterized by a central, triple-stranded, coiled-coil region, with outer carboxyl (C-) terminal antiparallel layers that are either mostly helical or adopt extended conformations, forming a trimer of helical hairpins. High-resolution crystallographic studies of the class I fusion glycoproteins from influenza HA[14], retroviruses (gp41 from human immunodeficiency virus [HIV][15], gp-41 from simian immunodeficiency virus [SIV][16], and gp21 from human T-lymphotropic virus[17]), and gp2 from Ebola virus[18] have all revealed similar structures[7,13]. Class I proteins commonly exist on virus particles as trimeric spikes that extend away from the particle membrane. The transmembrane anchor, the amino acid glycine-rich fusion peptide (varies from 10–30 amphiphilic amino acids), and the external folded domains all contribute to membrane fusion in the class I family.

Influenza HA is a prototype for the class I fusion proteins and is responsible for much of our present knowledge of viral membrane fusion. HA has been well characterized thanks to X-ray crystallography of three separate conformations relevant to the fusion process, which have guided many modeling efforts of membrane fusion[14]. Inspired by this detailed structural knowledge of HA, a *de novo* designed, coiled-coil system was recently created that has low-pH enhanced membrane fusion activity[19].

Influenza particles, like all type I fusion protein–containing viruses, are pleomorphic with most particles being spherical or oval, and having length scales of 100–200 nm. On average, influenza particles contain around 375 HA spikes that extend approximately 12 nm out from the viral membrane surface[20]. The single chain precursor, HA0, cleaves into HA1 and HA2 on activation[14]. HA1 and HA2 compose the metastable neutral pH conformation of HA found on mature virus, i.e., the prefusion conformation. Terminal sialic acids of glycoproteins and glycolipids (i.e., gangliosides) are the cellular receptors for influenza. The hydrogen bonds and van der Waals contacts between sialic acid and the carbohydrate side chains of HA1 bind influenza to the cell at neutral pH[21,22,23]. These receptor-bound particles are endocytosed. Acidic conditions are encountered along the endosomal transport pathway, at which point membrane fusion is triggered between the influenza particle and the endosome[24].

Several crystal structures of HA under different conditions, along with biochemical studies, have guided formulation of a model of the structural dynamics that lead to membrane fusion. A loop-to-helix transition in HA2 is thought to project the fusion peptide 100 Å towards the endosomal membrane where it anchors irreversibly, thus creating a metastable, extended conformation in which both membranes are cross-linked by HA. Next, HA2 performs a reverse turn and jackknifes back, relocating the C-terminus by 100 Å towards the target membrane, thus bending the molecule in half like a hairpin (Fig. 1)[25]. The C-terminal, $\alpha$-helical coil interacts in an antiparallel fashion with the N-terminal coil. In this way, the three HA proteins comprising a surface spike assemble into a “trimer of hairpins”, where the transmembrane domain and fusion-peptide segments of each protein are together at the same end of the molecule. With the N- and C-terminus now adjacent to each other, membrane fusion can occur. This fusion-pH conformation is the lowest free-energy state characterized for HA2[26].

As of yet there is no consensus on what conformations and entry angles of HA are necessary to destabilize the lipid membranes sufficiently to activate fusion. Some studies suggest that only one HA trimer is necessary for binding and fusion, but other studies suggest that a complex of three or six trimers is required[27,28,29]. Also, the number and form of intermediate structures is still under debate[14].

Although all class I fusion proteins are thought to follow the general sequence of conformational changes seen in HA, not all of them use endocytosis to enter the cell or are triggered by exposure to acidic conditions. HIV, avian sarcoma/leukosis virus (ASLV), Sendai virus, and murine leukemia virus (MLV) all fuse directly to the plasma membrane at neutral pH[30,31]. Fusion of HIV-1 is triggered by the interaction of the virus fusion proteins with a receptor and a coreceptor[32]. HIV-1 glycoprotein 120 (gp120) binds to the cell receptor CD4, usually found on helper T cells. Gp120 is loosely bound to gp41, which is embedded within the viral membrane[33]. A C-terminal antiparallel $\beta$-sheet forms the base of a trimeric protein spike. When HIV-1 binds to a cell, the interaction of gp120 with CD4 triggers a transition in gp120, allowing gp120 to interact with the secondary cell-surface receptors CXCR4 or CCR5[34,35]. Fusion between the viral membrane and the cellular membrane proceeds following an additional conformational change in gp41

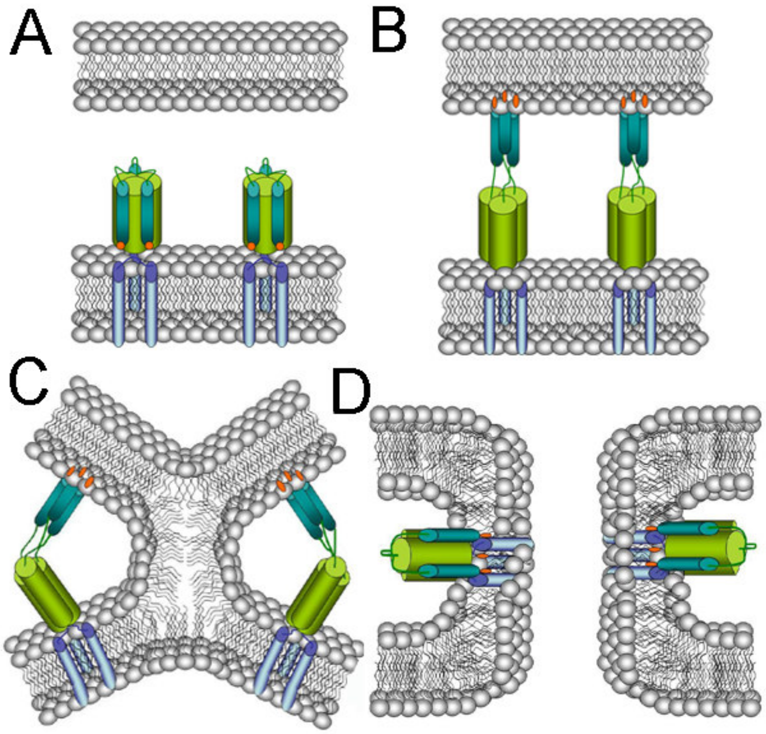

**FIGURE 1.** Conformational changes in influenza HA protein related to membrane fusion. (A) The trimeric membrane fusion protein spike assembly of influenza virus is anchored into the viral membrane in a preactivated state. Shown schematically, the transmembrane domain is dark blue, the N-terminal fusion peptide is orange, the green cylinders depict α-helical structures. On acidification, a hypothesized "extended intermediate" conformation is generated in which the hydrophobic fusion peptide is extended towards the targeted membrane. (B) After anchoring into the endosome's membrane, to cross-link the two membranes, the fusion protein folds back into a hairpin, positioning the transmembrane domain and fusion peptide in close proximity. (C) This hairpin forces the two membranes close together and is essential for the process, leading to full merging of the bilayers. (D) A fusion pore forms when the C- and N-terminus are relocated adjacent to each other in the same membrane[62].

that results from the secondary receptor interactions[36]. Gp41 at low surface concentrations promotes fusion, whereas it leads to rupture at high surface concentrations[33]. Similar to HA, conformational changes in gp41 expose an N-terminal fusion peptide, which will insert into the cell membrane. The ability of short peptides (C34 and T-20, or enfuvirtide) to bind and stabilize an elongated form of gp41, inhibiting overall fusion, is the strongest evidence for the existence of an extended intermediate conformation for all fusion proteins on the pathway to membrane fusion[32,37,38,39,40,41,42]. Despite HIV's standing as the prototype for viruses that fuse at the plasma membrane, very recent experiments have challenged this view[43]. These recent studies found substantial evidence that membrane fusion within an endocytotic pathway is the dominant cell entry mechanism leading to productive infection for HIV.

## Class II Fusion Proteins

Class II proteins are found in alphaviruses (E1 from Semliki Forest virus [SFV][44] and Sindbis virus [SIN][45]) and flaviviruses (E1 from hepatitis C virus [HCV][46,47,48]; E from tick-borne encephalitis [TBE][49], dengue fever[50], and West Nile virus[51]). Class II proteins are expressed as a single polypeptide precursor that is cleaved prior to the virus' release from the infected cell and long before entering the endocytotic pathway. The cleavage of p62 yields E1 and E2 in alphaviruses, and the cleavage of prM yields E and M in flaviviruses. HCV envelope proteins are not matured by a cellular endoprotease cleavage[46]. The E and E1 glycoproteins fold into three domains primarily composed of β-sheets[52].

Alphaviruses enter the cell via endocytosis[53], as shown by their ability to fuse with liposomes at low pH[54], although some evidence suggests alternate entry pathways[55,56]. SIN particles have 240 copies of E1, capsid, and E2. E1 and E2 form heterodimers with mostly β-barrel structures that are linked together to form an icosahedral structure (T = 4 quasisymmetry) with an external diameter of 70 nm. The icosahedral symmetry of class II proteins containing viruses is a key distinction from the class I protein containing viruses, but is similar to many nonenveloped viruses that do not contain membrane fusion proteins. Remarkably, recent structural investigations have suggested that despite the dramatically different initial conformations, the mechanisms of mediating membrane fusion appear nearly identical for class I and class II proteins[6,7,13,26,45,57]. Functional studies support this conclusion of similar fusion mechanisms for class I and II proteins[58,59].

E1 is responsible for cell fusion, and E2 is involved in receptor binding and cell entry. E1's fusion peptide lies near the dimerization domain, having many contacts with E2. Fusion requires the dissociation of the tightly packed E1-E2 heterodimers, exposure of the fusion peptide, and the formation of E1 homotrimers. On exposure to low pH, E1-E2 heterodimers rotate, moving the bulky E2 molecules out of the center of the prefusion assembly and allowing E1 homotrimeric spikes to form. These E1 spikes extend away from the virus surface. Because the E1 fusion protein is a major assembly element of the icosahedral prefusion lattice, these movements result in a major rearrangement of the viral surface.

The fusion peptide is located at the tip of the extended E1 spike, allowing it to embed in a targeted membrane. In the final postfusion state, the E1 molecule is bent at the central "hinge" domain, which is nearly centered between the fusion peptide and the C-terminal membrane anchor. This final conformation suggests that dynamic bending at the hinge allows E1 to transition from an extended conformation, cross-linking two membranes to a jackknifed conformation, bringing the viral membrane and the snared target membrane close together[26,44,50,52,57,59,60,61,62], similar to the dynamic motions in influenza HA and HIV gp41. Cryoelectron microscopy revealed that the E1 transmembrane domain becomes bent and does not completely traverse the membrane in the final postfusion state, suggesting a role for transmembrane domain–induced stress in the bilayers during fusion[57].

## Class III Fusion Proteins

Class III proteins combine structural elements from both class I and class II fusion proteins. The fusion domain in class III proteins contains two fusion loops at the tip of an elongated β-sheet similar to the class II proteins. However, the postfusion state is similar to the α-helical, hairpin-like trimeric core seen in class I proteins[13,63]. Baculovirus gp64[64], vesicular stomatitis virus (VSV) G[65], and herpes simplex virus type 1 (HSV-1) gB[66] proteins are suggested to be class III proteins.

For most fusion proteins, a single trimer refolding releases more free energy than is required to overcome the barrier to fusion. Rhabdoviruses are unusual in that the energy available from refolding one trimer is insufficient to catalyze fusion[63,67]. Therefore, for this virus family, multiple fusion trimers are required to associate before fusion can occur[68].

## Similarities to SNARE Proteins

Intriguingly, some of the same structural features seen in the viral fusion proteins are also present in the eukaryotic cellular fusion proteins, the SNAREs[1]. SNARE proteins are present in all eukaryotic cells on multiple compartments and mediate fusion between cellular transport vesicles, e.g., synaptic vesicles and the cell membrane[69]. SNAREs are membrane proteins anchored to membranes by either single-pass transmembrane domains or palmitolyation. The heteromeric complex formed from SNARE proteins (the SNARE complex) is composed of two transmembrane-containing SNAREs that bind to each other along with additional SNARE proteins and additional factors (e.g., Sec9, Munc18, complexin, etc.). Formation of this complex brings two apposing membranes together in a manner very similar to viral membrane fusion proteins[70,71,72,73]. On complex assembly, the transmembrane domains of SNAREs in each membrane cross-link the membranes and positions them at the same end of a coiled coil of helicies[74], similar to the final configuration of viral fusion proteins with the transmembrane domain and fusion peptides closely positioned in the jackknifed, folded-back conformation. When complementary sets of SNAREs are anchored in opposed membranes, complex assembly provides the energy necessary to connect these membranes and overcome the repulsive forces that would otherwise prevent fusion[75]. The precise mechanisms connecting SNARE protein folding and protein-lipid interactions to membrane fusion continue to be open questions. Nevertheless, the similarities in structures between SNARE complexes and viral fusion proteins when cross-linking membranes destined to fuse suggest that the mechanism of achieving membrane fusion is common.

## THE ROLE OF LIPIDS IN MEMBRANE FUSION

Membrane fusion proteins ensure close apposition of the membranes during fusion phenomena; yet, simply forcing membranes into close proximity is not sufficient to yield membrane fusion. Protein-free bilayers will not spontaneously fuse with each other even when separated by only 2–3 nm[8,76], which demonstrates the crucial role of interactions between fusion proteins and lipids for fusion. The presence of certain lipid species and lipid derivatives within the bilayers can also strongly promote or inhibit fusion, likely by modulating the spontaneous curvature of bilayers[31,77,78,79]. Lipid composition may also be critical for recruitment of other proteins to the plasma membrane, organization of membrane domains for fusion, and regulation of fusion protein complexes[78].

Cellular plasma membranes contain many different classes of lipids with different molecular shapes and charge distributions, including phosphatidylcholine (PC), phosphatidylserine (PS), phosphatidylinositol (PI), phosphatidylethanolamine (PE), cholesterol (CH), and sphingomyelin (SM). CH and sphingolipids form lipid raft domains within the lipid bilayer[80,81]. These lipid raft domains are involved in the entry, assembly, and budding of viral structural proteins for some enveloped viruses. Lipid raft domains influence cell entry for influenza, Ebola, Marburg, Epstein-Barr, and HSV-1[21,35]. HA binds to sialic acid moieties present on glycolipids that are often clustered in rafts within the cell membrane. Interactions between the transmembrane domain and lipids cause HA to concentrate in lipid rafts[35]. Flavivirus fusion is enhanced by CH; however, CH is not absolutely required for fusion[26]. SFV and SIN require the sterol 3β-hydroxyl group in CH for cell binding and entry; however, they do not require lipid rafts for fusion[31,79]. E1's binding and acid-dependent conformational changes are both inhibited by a lack of CH in the target membrane[58,82,83].

*In vitro* experiments using carefully designed blends of specific lipids can arrest fusion at a specific stage or inhibit fusion altogether, providing critical insights into the fusion process[84,85]. Inverted cone-shaped lipids, such as PS or lysophosphatidylcholine (lysoPC), promote positive spontaneous curvature in the bilayer. These lipids inhibit fusion when present in the outer monolayers of membranes, but promote fusion between these same membranes' inner monolayers by enhancing formation of a fusion pore. Conversely, lipids that promote negative spontaneous curvature in the bilayer, such as PE, enhance fusion between outer monolayers, but arrest fusion between inner monolayers, leading to a hemifusion structure that resembles a stalk[8,86,87].

This stalk structure has been observed in a static configuration between bilayers composed of diphytanoylphosphatidylcholine (DPhPC)[88,89]. For that observation, the bilayers were first brought into contact by dehydration. Further reduction of the intervening water resulted in formation of stalks at the interbilayer contact points. In viral membrane fusion experiments where tracers have been placed both in the membrane as well as within the aqueous compartment of the target structure, it has been shown that outer lipid monolayer leaflets exchange molecules before aqueous compartments merge (Fig. 2), which suggests that the stalk forms before the fusion pore opens[27,54,89,90,91,92]. No consensus on a model of the organization of a fusion protein–containing lipid hemifusion stalk has been reached.

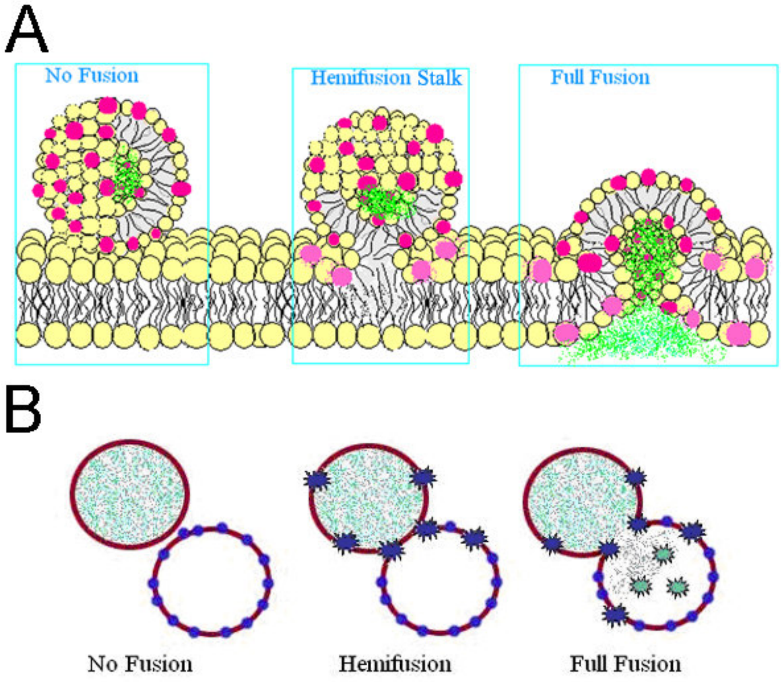

**FIGURE 2.** Fluorescent lipid-like dyes incorporated into a virus membrane can report on lipid mixing occurring during hemifusion, but detection of content mixing is required to confirm pore formation and full fusion. (A) Fusion of a vesicle with membrane and content dye to an unlabeled planar bilayer. (B) Fusion of a membrane-labeled vesicle to a content-labeled vesicle.

The free energy of the stalk structure is dependent on its geometry and the properties of the lipid monolayer[88,89]. The bending of bilayers away from their preferred spontaneous curvatures is resisted by the required changes in both the packing density of the lipid head groups on the surface and the lipid acyl chains within the hydrophobic core of the bilayer (see Fig. 1C)[77]. Prior to forming a fusion pore in a stalk, the inner monolayers may pucker inward and touch, a state called transmembrane contact (TMC). The TMC may then expand radially outward forming a hemifusion diaphragm (HD) (Fig. 3)[76]. Another possibility is that partial insertion of the fusion loops and fusion peptides into the outer leaflet of the target membrane deform the bilayers. This partial insertion could displace the lipid head groups on the outer leaflet, resulting in a difference in area between the inner and outer leaflet, thus causing the bilayer to curve into a nipple-like cap. For influenza, the formation of a cap on the HA2 helical bundle would make pore formation irreversible[7,87,93].

Tension or compression in the bilayers can be exasperated or relieved by osmotic gradients. A compressive (negative) osmotic gradient will press lipid material from the inner or outer leaflet towards the void, promoting pore formation. A swelling (positive) gradient has the opposite effect, pressing lipid material from the void toward the outer leaflet, inhibiting pore formation. Lipids with natural tendencies toward membrane curvature, either positive or negative, also influence the energy balance in these intermediate structures. Some conditions in a hemifused stalk can generate an unstable, flickering fusion pore that will either open fully, allowing for the exchange of material between aqueous compartments, or collapse[76,94]. It is not absolutely necessary for proteins to participate in hemifusion stalks or fusion

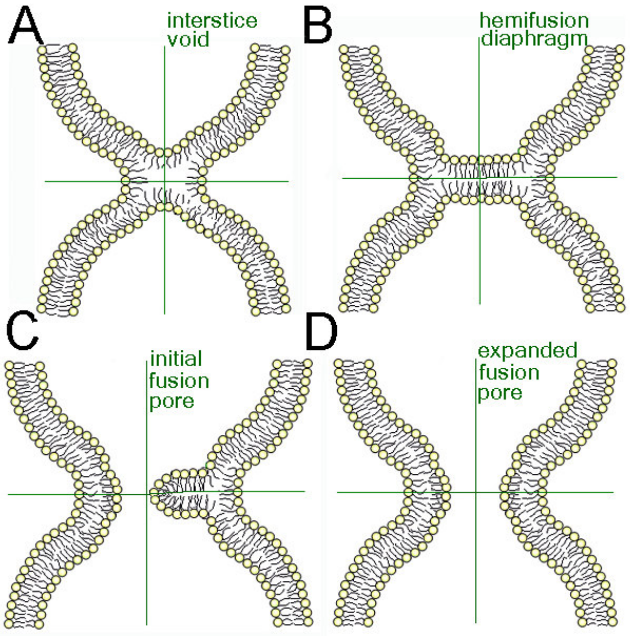

**FIGURE 3.** Hemifusion diaphragm (HD) in membrane fusion. (A) Voids in the junction of fusing membranes create steep energy barriers. (B) Membrane tilt and formation of HD lower the energy barrier. (C) Formation of a fusion pore begins when the HD's radius expands beyond the membrane's thickness generating a rupture near the HD's rim. This fusion pore may flicker in and out of existence before becoming stable. (D) As the HD continues to expand, the pore becomes large enough for the RNA/DNA to pass through it[11,76].

pores, as evidenced by the fact that pores are seen in protein-free liposome systems where poly(ethylene glycol) and dehydration were used to fuse the vesicles without a hemifusion intermediate[95].

A direct role for the transmembrane domain in the mechanism of membrane fusion has been revealed by experiments using engineered HA in which the transmembrane domain has been replaced by a glycosylphosphatidylinositol (gpi) lipid anchor. In these experiments without the transmembrane domain, membrane fusion is arrested at the hemifusion state with no fusion pore forming even after very long times. Whether the transmembrane domain serves to couple forces created by protein folding into the bilayer, disrupts the lipid packing, or contributes structurally to the transmembrane pore remains a point of debate[96,97,98,99].

The actions of the fusion peptide on the lipid bilayers are as important to the overall membrane fusion process as the role of the lipids and the lipid-transmembrane domain interactions. Mutations in the short fusion peptide have a very large effect on the efficiency of fusion[100,101,102,103,104]. A mechanistic understanding of how fusion peptides promote fusion is incomplete despite a number of theories about modes of action including curvature induction, membrane rupture promotion, or direct force transduction[105].

Cooperative interactions of the membrane-contacting domains of the fusion proteins may also be fundamental to membrane fusion. Baculovirus gp64 requires associations between viral proteins for membrane fusion. For other fusion proteins, cooperativity is likely, although not energetically, necessary. The possibility of protein interactions led to speculation that a ring-like complex of glycoproteins around the hemifusion stalk might restrict lipid flow between the bilayers. If proteins form a loosely packed ring around the TMC, then lipids will be able to mix within the stalk, allowing for lipid mixing without content mixing (Fig. 4). If proteins are packed tightly in a ring around the TMC, then lipid flow will be restricted between outer leaflets and content mixing may precede lipid mixing[9,67]. Protein cooperativity

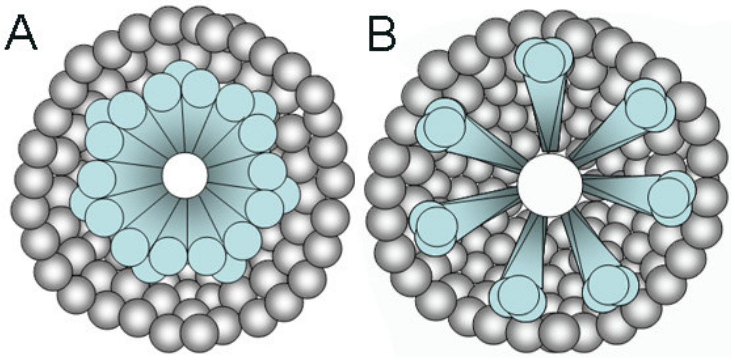

**FIGURE 4**. Top view of two models of details of the fusion pore in a lipid bilayer. Proteins are the light blue and lipids are the gray circles. (A) Lipids are prevented from mixing between the two membranes by a tight collar assembled from several fusion proteins. (B) The assembly of transmembrane domains of multiple fusion proteins at the fusion pore is loose enough to allow lipids to diffuse between the two membranes[28].

has also been suggested for other membrane fusion protein phenomena, including SNARE-mediated fusion[106]. In the SNARE system, the energy required for hemifusion between outer leaflets was experimentally determined to be 1.4 kJ/mol. The same SNARE experiment determined that pore formation between inner leaflets required 5.5 kJ/mol[70]. Extrapolating this result to general membrane fusion confirms that the energy required for pore formation is much larger than the energy required for hemifusion, and may suggest that cooperative protein interactions could assemble after hemifusion in order to advance the evolution of the stalk toward pore formation efficiently[76].

## EXPERIMENTAL TECHNIQUES THAT REVEAL DYNAMICS OF MEMBRANE FUSION

The importance of X-ray crystallography to our understanding of membrane fusion cannot be overstated. Landmark structures of HA, E1, gp41, and gp120 in a variety of pre- and postactivated states have not only given direction to the field of membrane fusion, but were also critical milestones in the field of structural biology[15,18,25,49,50,51,107,108,109]. Electron cryomicroscopy and X-ray crystallography have provided insights into the overall virus particle composition[20,45,104,108,110]. Both techniques can achieve near Å resolution, but provide only static images[111]. Even with images such as the pre- and postfusion configuration of HA, the actual motions and refolding of the protein during the membrane fusion process are unknown. Dynamic measurements of membrane fusion are required to complete our understanding of membrane fusion.

Conductivity measurements have been used to observe dynamic formation of the fusion pore in lipid bilayers and biochemical studies have provided information about the kinetics of the actions of the fusion proteins. Incorporation of fluorescent labels into the virus particles has allowed the most detailed studies of the time course of membrane fusion. For these measurements, fluorescent lipid-like markers are added to the lipid bilayer of viruses, cells, and liposomes, or aqueous fluorescent labels are added to the interior of the compartments. Often, these fluorescent labels are added at high enough concentrations to self-quench, so that on fusion, they mix into the target compartment and dilute, relaxing the quenching and increasing the fluorescence[83,90]. Variations of this assay use resonant energy transfer between two distinct fluorophores to report concentration[112]. Bulk fusion studies using this method have helped determine the lipid composition, pH conditions, and temperature dependencies of virus-membrane binding and fusion[22,29,33,54,83,90,113,114,115].

Greater details of the kinetics of membrane fusion intermediates can be gleaned by using a microscope capable of imaging the fluorescence of individual dye-labeled virus particles. For example, the single-particle approach has directly revealed the times between binding and fusion (when no hemifusion intermediate is detectable), between binding and hemifusion, and between hemifusion and full fusion[23,27,28,58,94]. A recent single-particle fluorescence study of individual influenza particles fusing under acidic conditions with lipid bilayers has discovered the existence of about three intermediate steps between low-pH activation and the quasistable hemifusion stalk state for particles bound to membranes at neutral pH through receptor interactions[27]. One further single kinetic step was required to proceed from hemifusion to opening of the fusion pore. A different study that examined single influenza virus particles exposed to low pH before encountering the lipid bilayer found a single step process to hemifusion[58]. Comparing experimental protocols of this sort may allow distinct intermediates in the fusion process to be teased apart. The success of these examples suggests that the single-particle approach holds great promise for future studies of membrane fusion.

The single-particle fluorescent microscopy imaging techniques can be adapted to a number of different configurations. By using streptavidin-coated surfaces, individual biotinylated vesicles can be immobilized on surfaces for imaging[116,117]. The presence of the surface may affect the free energy of the system[118], but the ability to make extended observations of a single virus-liposome complex provides an approach that can overcome the limitations of bulk fusion assays due to the stochastic nature of each fusion event and the difficulty in synchronizing fusion of large ensembles of particles. Liposome targets also allow the role of membrane curvature to be tested in fusion experiments. With all fluorescent labeling experiments, careful consideration of the potential artifacts due to the presence of the label or products of photobleaching the dye labels is important[115]. Interferometric optical detection appears to be a promising technique that is photostable, allows for long time measurements and fast tracking, and leaves the biological system unperturbed. Presently, this technique is difficult to apply within an intact cell[119].

The single-particle fluorescence tracking approach has been used to observe individual virus particles interacting with living cells (for reviews, see [5,120,121,122,123]). Protein dye labels, membrane dye labels, and green fluorescent protein (GFP) have been used to track viruses as they enter the cell and after entry into the cell as they moved towards the nucleus[124,125].

For enveloped viruses, labeling the viral membrane with self-quenched concentrations of fluorescent dye has allowed membrane fusion with cellular membranes to be detected during single-particle tracking experiments[126,127,128,129]. For some viruses, the moment of membrane fusion has been correlated with a sequence of well-defined active transport steps following endocytosis that move the virus to specific locations in the cell[126,129,130]. Double labeling of viruses with pH-sensitive dyes demonstrated that endosomal acidification precedes fusion[129,131]. In influenza, single-particle tracking revealed that fusion occurs during late endosomal acidification near the perinuclear region of the cell typically about 8 min following the initial binding of the virus to the cell[129]. Single-particle tracking techniques have been used to show that for HIV-1, aqueous contents never transfer before the spread of lipid dye. The time between lipid dye spread and aqueous content transfer is stochastic, sometimes occurring several minutes after the membrane label has disappeared, and is dependent on the target cell type[128]. Recent single-particle tracking of HIV in live cells has concluded that content release only follows lipid mixing when viral particles are endocytosed[43]. This paradigm shifting result demonstrates that measuring biophysical behaviors of viruses in the context of their interactions with living cells can be essential in order to understand their full physiological implications.

## MOLECULAR DYNAMICS (MD) SIMULATIONS

With the current limitations on gathering dynamic experimental data at the nanometer scale with time resolutions under a microsecond, efforts in theoretical and computational modeling have been used to address the dynamics of membrane fusion. These theoretical/computational efforts are challenged by the complexity of this phenomenon, which includes protein folding, lipid rearrangement, dependence on the aqueous environments, and possible cooperativity between proteins.

Historically, phenomenological modeling of membranes as elastic materials were the dominant theoretical efforts to model membrane fusion and revealed the general aspects of the hemifusion stalk-diaphragm model[8,9,62]. More recently, MD simulations are addressing the dynamic transitions during membrane fusion. Simulations examining the lipid membrane show the formation of stalks between the diamond cubic phase (QII) to an inverted hexagonal phase (HII)[132]. In these simulations, transmembrane connections precede stalk formation. Forces that tend to minimize the interface curvature affect the transitions between the states. Voids that arise when three monolayers merge can have large free-energy penalties, but these simulations suggest that the lipids tails are flexible enough to fill the bilayer where the monolayers connect[132]. Another MD simulation examined rearrangements when lipids are randomly removed from one monolayer. When more than 12 lipids are removed, the lipids in the intact monolayer will stretch to cover the exposed chains, suggesting a mechanism to generate bending forces in the bilayers by manipulating local lipid density[133].

A course-grain MD simulation of two vesicles fusing due to cross-linker tethering has shown membrane fusion between a pair of 14-nm-diameter vesicles on the hundred-microsecond time scale. The cross-linker is analogous to the prefusion states of HIV gp41, influenza HA, or trans-SNARE complexes. The simulation showed fusion proceeding via a stalk-like intermediate state. About 20% of vesicles rapidly fuse from the stalk state. The remaining vesicles remained in a long-lived hemifusion intermediate that slowly converted to the fused state. The hemifused intermediate state had a decay half-life of 6.3 μsec. In addition to being able to measure $t_{1/2}$, the free energy of the stalk-like state ($\Delta G$ of −3.3 kcal/mol) and fused state ($\Delta G$ of −6.0 kcal/mol) were determined. However, a determination of the energy of fusion pore expansion did not result from this simulation[134].

Simulations of atomic force microscopy measurements of the cowpea chlorotic mottle virus (CCMV) capsid provided a test bed for modeling forces in the capsid shell lattice that can couple the response of the large structure to local conformational changes[135]. Although the capsid is not involved in membrane fusion for most viruses, similar experiments and analysis of whole alpha- and flavivirus particles might advance our understanding of the large-scale lattice rearrangements of the network of fusion proteins that are present in these highly symmetric particles.

## ANTIVIRALS

Knowledge of the fundamental biophysical properties of viruses can guide research into development of antiviral treatments. For example, understanding of the functional role of the ion channel M2 and the enzyme neuraminase from influenza have allowed us to learn the mode of action of current anti-influenza drugs[136]. Membrane fusion is a basic function important to many viruses that can also be targeted for drug development. Recently, tert-butyl hydroquinone (TBHQ), a known inhibitor of membrane fusion, has been proposed to treat influenza infection. When TBHQ binds in a hydrophobic pocket formed at an interface between HA monomers, it stabilizes the neutral pH structure of HA, inhibiting the conformational rearrangements required for membrane fusion[109]. Similarly, antibodies that do not prevent cell binding can inactivate influenza by preventing conformational changes in HA that are necessary for membrane fusion[137].

Many of the current antiviral drugs for HIV target reverse transcriptase and HIV Gag protease. Viruses quickly become resistant to these drugs, so an inhibitor that will block the binding between CD4 and HIV's gp120 has been sought. Enfuvirtide (T20) is an FDA-approved antifusion drug that is suggested to interfere with gp41, but viral mutations in the binding site of this drug can lead to decreased efficacy[138,139,140]. C34 is a synthetic peptide that also inhibits fusion between HIV and the target cell by binding to the intermediate conformation of gp41, preventing formation of the six-helix bundle structure necessary for fusion[39,141,142]. Additional experimental HIV fusion inhibitors include, but are not limited to, T-2635, T-651, and bifunctional fusion inhibitors (BFFI)[143], which are generated by attaching fusion inhibitors to a CCR5 or CD4 antibody[144]. Complementary approaches focus on blocking the second binding step of gp120 to the cell's coreceptor CCR5 or CXCR4. Naturally occurring anti-CCR5 antibodies have been found that protect against and help control the HIV infection. Already the antiviral PRO-140 has been clinically proven to inhibit binding between CCR5 and gp120. Due to the multistep procedure HIV uses for cell entry, it has been proposed that a CCR5 antibody covalently linked to a fusion peptide inhibitor like enfuvirtide may prevent HIV entry into the cell at multiple steps[143].

The antigenic nature of viruses, variety of viral binding sites, and high virus mutation rates make it difficult for most drugs to remain effective against viruses over time. However, many enveloped viruses employ similar entry mechanisms that require fusion between the viral envelope and cell/endosomal membrane. If a therapeutic strategy is effective against a key biophysical property of membrane fusion, then perhaps drugs targeting this mechanism will remain more effective over time. The commonality of membrane fusion mechanisms among enveloped viruses may allow for similar therapeutic strategies to be employed against many enveloped viruses[141].

## CONCLUDING REMARKS AND FUTURE DIRECTIONS

The study of viral membrane fusion has provided insight into a fundamental process that occurs widely in eukaryotic cell biology. The functional commonality among class I, II, and III viral fusion proteins is surprising considering the diversity of structures and the varied fusion triggers. The current model of viral membrane fusion proposes that a cellular trigger initiates a conformational change in the viral fusion proteins, which exposes a hydrophobic viral fusion peptide. This peptide extends outwards towards and inserts into an apposing cell membrane. The fusion protein then folds back into a hairpin to bring the two apposed membranes together. The subsequent fusion of these membranes is dependent on the lipid composition of the membranes as well as possible additional protein conformational changes and cooperativity among several fusion proteins. The exact mechanisms that lead to lipid mixing are not known, but the precise sequence of the fusion peptide along with the transmembrane domain of the viral fusion protein is indispensable. It is believed that an intermediate hemifusion stalk is formed that then expands into a diaphragm membrane structure. Finally, a fusion pore forms within the diaphragm. The created fusion pore may be stable or may flicker repeatedly before expanding into an opening large enough to allow the genetic material of the virus to enter the cell. The exact nature of these intermediate structures is unknown. Future studies are needed to determine how viral fusion proteins and membrane lipids interact to generate a membrane instability that is large enough to produce membrane fusion.

## ACKNOWLEDGMENTS

The research of KW is supported in part by a CASI award from the Burroughs Wellcome Fund. We thank John Sakon for critically reading the manuscript.
